# Hypertension in pregnancy and risk of coronary heart disease and stroke: A prospective study in a large UK cohort

**DOI:** 10.1016/j.ijcard.2016.07.170

**Published:** 2016-11-01

**Authors:** Dexter Canoy, Benjamin J. Cairns, Angela Balkwill, F. Lucy Wright, Asma Khalil, Valerie Beral, Jane Green, Gillian Reeves

**Affiliations:** a Cancer Epidemiology Unit, Nuffield Department of Population Health, University of Oxford, Richard Doll Bldg., Roosevelt Drive, Oxford OX3 7LF, UK; b Fetal Medicine Unit, Department of Obstetrics and Gynaecology, St. George's Medical School, University of London, London, UK

**Keywords:** Hypertension, Hypertensive pregnancy, Women, Coronary heart disease, Stroke

## Abstract

**Background:**

Many studies investigating long-term vascular disease risk associated with hypertensive pregnancies examined risks in relatively young women among whom vascular disease is uncommon. We examined the prospective relation between a history of hypertension during pregnancy and coronary heart disease (CHD) and stroke in middle-aged UK women.

**Methods:**

In 1996–2001, 1.1 million parous women (mean age = 56 years) without vascular disease at baseline reported their history of hypertension during pregnancy and other factors. They were followed for incident CHD and stroke (hospitalisation or death). Adjusted relative risks (RRs) were calculated using Cox regression.

**Results:**

Twenty-six percent (290,008/1.1 million) reported having had a hypertensive pregnancy; 27% (79,163/290,008) of women with hypertensive pregnancy, but only 10% (82,145/815,560) of those without hypertensive pregnancy, reported being treated for hypertension at baseline. Mean follow-up was 11.6 years (mean ages at diagnosis/N of events: CHD = 65 years/N = 68,161, ischaemic stroke = 67 years/N = 8365, haemorrhagic stroke = 64 years/N = 5702). Overall, the RRs (95% confidence interval [CI]) of incident disease in women with hypertensive pregnancy versus those without such history were: CHD = 1.29 (1.27–1.31), ischaemic stroke = 1.29 (1.23–1.35), and haemorrhagic stroke = 1.14 (1.07–1.21). However, among women with hypertensive pregnancy who were not taking hypertension treatment at baseline, their RRs (95% CI) were only modestly increased: CHD = 1.17 (1.14–1.19), ischaemic stroke = 1.18 (1.11–1.25), and haemorrhagic stroke = 1.09 (1.02–1.18).

**Conclusion:**

Hypertension during pregnancy was associated with increased CHD and stroke incidence in middle age, largely because such women also had hypertension in their 50s and 60s, which has a substantially greater effect on vascular disease risk than hypertension during pregnancy without hypertension later in life.

## 1 Introduction

Hypertension during pregnancy is an important cause of morbidity and mortality in both the mother and her offspring during the perinatal period [1], [2], [3]. Its effect on the mother's vascular health has been reported to extend for many years after the pregnancy [4], [5], [6], [7], [8], [9], [10], [11], [12], [13], [14], [15], [16], but many studies have been in relatively young women among whom vascular disease rates are still relatively low [17], [18]. There is limited information on the impact of hypertension in pregnancy on the vascular health of women in middle age [7], [10], [12], [15], [16] when vascular disease rates are relatively high [17], [18] and unhealthy lifestyles and other factors, such as body mass index, diabetes, dyslipidaemia, and hypertension, become important determinants of coronary heart disease and stroke in the population [19], [20]. Furthermore, separate risks for ischaemic and haemorrhagic strokes remain unclear. We studied the relationship between a history of hypertension during pregnancy and subsequent risks of coronary heart disease and ischaemic and haemorrhagic stroke in a cohort of over a million UK women. We explored these associations in middle-aged women, taking into account their health and lifestyle and other risk factors of vascular disease.

## 2 Methods

The Million Women Study is a population-based prospective study which recruited 1.3 million UK women aged 50 to 64 years who were invited to routine breast cancer screening programmes of the National Health Service (NHS) in England and Scotland from 1996 to 2001. The study design and recruitment have been described in detail previously (www.millionwomenstudy.org) [21]. At recruitment, women filled in a questionnaire which included questions on reproductive and medical history, weight, height, lifestyle, and socio-economic indicators. The study population included about 1 in 4 of all UK women aged 50 to 64 years at the time of recruitment. Participants gave written consent to take part in the study and the Oxford and Anglia Multi-Centre Research Ethics Committee gave ethical approval to the study. We linked with NHS data sources for hospital admissions and mortality to obtain information on hospitalisations and vital status for the whole cohort [22], [23]. The NHS hospital admissions data for in- and day-patients have been obtained through linkage with the Hospital Episode Statistics [24] for participants in England and the Scottish Morbidity Records [25] for participants in Scotland. Hospital diagnoses and causes of death are reported to the study coded to the World Health Organization's International Classification of Diseases, Tenth Revision (ICD-10) [26].

At recruitment (baseline for this study), women reported whether or not they ever had high blood pressure during pregnancy, and whether or not they were currently using any medication for selected conditions including hypertension, diabetes, and hypercholesterolaemia. Taking medications particularly for hypertension is a proxy measure for having had a clinically relevant elevation of blood pressure. Women provided information on reproductive factors including age at menarche, parity, and use of hormone replacement therapy. They also reported their smoking habits, alcohol consumption, frequency of strenuous physical activity, and socioeconomic status, as well as their weight and height, from which we calculated body mass index (BMI) (weight in kg/[height in m]^2^).

We defined incident vascular disease as the first hospital admission with any mention of vascular disease, or, where there was no relevant hospital admission, death with the vascular disease as the underlying cause. We identified a first vascular disease outcome separately for coronary heart disease (ICD-10: I20 to I25), cerebrovascular disease (ICD-10: I60 to I69), ischaemic stroke (ICD-10: I63), and haemorrhagic stroke (ICD-10: I60 to I62). Virtually all residents in the United Kingdom are registered with general practitioners who hold comprehensive information about an individual's health and medical care in the NHS. We previously reported that there was excellent agreement between vascular disease diagnoses recorded in hospital records and those ascertained from the reports of general practitioners [27].

In a resurvey of the cohort about nine years after baseline recruitment, a randomly selected subgroup of women (N = 16,954) provided non-fasting blood samples for lipid profile assessment. Details of blood sample collection, storage and lipid assays have been reported previously [28]. Among this subgroup, some women (N = 3694) also had their blood pressure measured at local general practices.

### 2.1 Statistical analysis

Of the 1.3 million women recruited to the study, we excluded 120,905 (8.9%) who reported or had a hospital record of heart disease, stroke or cancer (except non-melanoma skin cancer) before recruitment. Of the remaining 1.2 million women, we further excluded 135,667 (9.9%) who were nulliparous and 2082 (0.2%) with missing data on parity. Data for 1,105,568 parous women with information on hypertension during pregnancy formed the basis of our analysis. We estimated hazard ratios, hereafter referred to as relative risks, of vascular disease incidence and mortality using Cox regression with age as the underlying time variable. Person-years were calculated from the date of recruitment until date of first hospitalisation, death, emigration and other loss to follow-up, or end of follow-up for this analysis (31 March 2011 in England and 31 December 2008 in Scotland), whichever came first. Around 5% of participants in England were recruited before 1 April 1997. Because hospital admissions data prior to this date were not available, we calculated their follow-up from this date.

All regression models were stratified by geographical region of recruitment (10 UK regions), and the covariates included body mass index (< 20, 20 to 24.9, 25 to 29.9, 30 to 34.9 and ≥ 35 kg/m^2^), smoking (never smokers, past smokers, and current smokers of < 5, 5 to 9, 10 to 14, 15 to 19, 20 to 24 and ≥ 25 cigarettes per day), socioeconomic status (fifths of Townsend index of deprivation [29]), frequency of strenuous exercise (rarely/never, once a week or less, and more than once a week), weekly alcohol intake (0, 1 to 6, 7 to 14 and ≥ 15 units), parity (1, 2, 3, 4, and ≥ 5 children), ever use of hormone replacement therapy (yes or no), and treatment for diabetes (yes vs no), hypercholesterolaemia (yes vs no), and hypertension (yes vs no) at baseline recruitment. Women with missing values for an adjustment variable were assigned to a separate category for that specific variable.

We calculated relative risks separately for each incident vascular disease outcome for all women and by women's use of anti-hypertensive medication at baseline. We also calculated the risks of death attributed to coronary heart disease and cerebrovascular disease. To examine further the consistency of the risks across different subgroups in the population, we examined the risks of incident coronary heart disease and stroke across a range of characteristics at baseline (socioeconomic status, parity, smoking, body mass index, vigorous exercise frequency, alcoholic beverage intake, hormone replacement therapy use, treatment for diabetes, and treatment for hypercholesterolaemia at baseline). For women with blood pressure readings and those with lipid profile data measured at around nine years after recruitment, we calculated the age- and region-adjusted mean systolic and diastolic blood pressure, as well as cholesterol and lipid fraction concentrations, according to women's history of hypertension during pregnancy and use of anti-hypertensive medication at baseline.

We report relative risks with their conventional 95% confidence intervals (CIs). When relative risks for multiple groups are presented (such as when estimating relative risks by joint categories of hypertension during pregnancy and being treated for hypertension in middle age), we report estimates with their 95% group-specific CIs [30] to allow direct comparison of risks between any two categories even if neither is the reference group. We examined heterogeneity in the associations of coronary heart disease and stroke across women's characteristics using likelihood ratio tests.

We used Stata 14.0 [31] programme to analyse our data, and used Jasper package version 2-210 [32] in R programme [33] to create the figure.

## 3 Results

In this analysis of 1.1 million parous women, the mean age at recruitment was 56.0 (SD = 4.8) years, and 26.2% (N = 290,008) reported that they had high blood pressure during pregnancy. Table 1 shows the characteristics of the participants at baseline. By far, the largest difference between those with and without history of hypertension during pregnancy was the proportion that reported being treated for hypertension at recruitment in their mid-50s: 27% (79,163 of 290,008) of those with a history of hypertensive pregnancy versus 10% (82,145 of 815,560) of those without such a history were on anti-hypertensive medication at recruitment.

Women were followed for 11.6 years (SD = 2.3 years) on average, accruing 12.8 million person-years of follow-up. Over this time period, 68,161 women had a first coronary disease event, 22,997 women a cerebrovascular disease, 8365 women an ischaemic stroke, and 5702 women a haemorrhagic stroke, with mean ages at first disease event of 65.0 (SD = 6.1), 66.2 (SD = 6.6), 66.9 (SD = 6.4) and 64.2 (SD = 6.6) years, respectively. Of these first vascular disease events, non-fatal hospital admissions accounted for 92% of coronary heart disease, 88% of cerebrovascular disease, 94% of ischaemic stroke, and 79% of haemorrhagic stroke. There were in total 7736 deaths attributed to coronary heart disease and 5554 to cerebrovascular disease, with mean ages at death of 65.9 (SD = 6.5) and 66.9 (SD = 7.3) years, respectively.

Compared to women without hypertension during pregnancy, the relative risk of a first coronary heart disease event for women with hypertensive pregnancy was 1.29 (95% CI 1.27 to 1.31) (Table 2). For coronary heart disease mortality, the relative risk was 1.35 (95% CI 1.29 to 1.42). The relative risks of a first cerebrovascular disease event or death due to this condition were also increased in women with hypertensive pregnancy. The risks of both ischaemic and haemorrhagic stroke were increased in women with hypertensive disease during pregnancy, with the relative risk of ischaemic stroke (1.29 [95% CI 1.23 to 1.35]) being slightly greater than the relative risk of haemorrhagic stroke (1.14 [95% CI 1.07 to 1.21]).

**Fig. 1:**
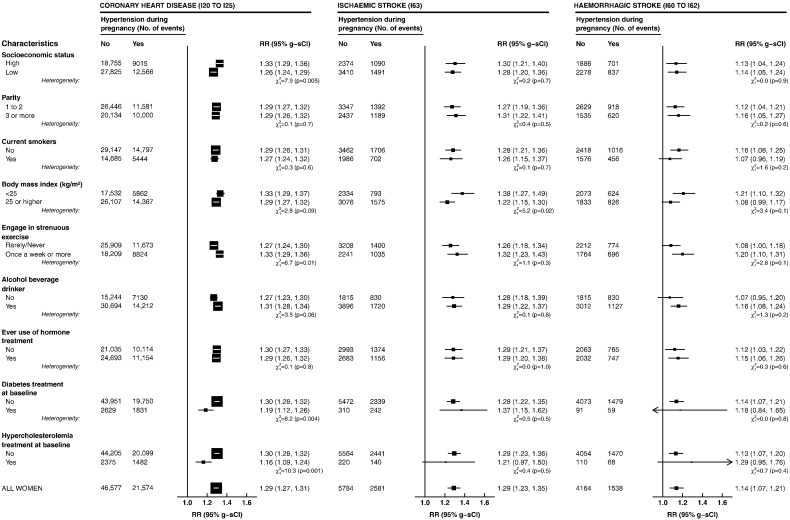
Relative risks (95% group-specific confidence interval) of incident coronary heart disease, ischaemic stroke, and haemorrhagic stroke in parous women with reported hypertension during pregnancy (N = 290,008) as compared to women without such history (N = 815,560), by their characteristics at baseline. Relative risks are hazard ratios estimated using Cox regression with age as the underlying time variable, stratified by region of recruitment, and adjusted for all listed factors except when the characteristic is under consideration. The area of the square is inversely proportional to the variance of the log risk. RR — relative risk; g-sCI — group-specific confidence interval; ICD-10 — International Classification of Diseases, 10th Revision.

Fig. 1 shows the relative risks of incident coronary heart disease and stroke across a number of subgroups as defined by the characteristics of women at recruitment. In all the subgroups, the risks of incident coronary heart disease and ischaemic stroke were consistently elevated in women with a history of hypertension during pregnancy. However, for incident coronary heart disease, the risks were somewhat weaker among women being treated for hypercholesterolaemia or diabetes. Nevertheless, these risks remained significantly elevated. We also observed a small increase in haemorrhagic stroke risks across various subgroups with no significant heterogeneity across any subgroup.

As many women who reported a history of hypertension during pregnancy also reported being treated for hypertension at baseline, we compared the risks in women categorised into subgroups as defined by these factors (Table 3). Compared with women with no history of hypertension in pregnancy or in middle age, at study baseline, women who reported treatment for hypertension (but no history of hypertension in pregnancy) had substantially raised subsequent risks of coronary heart disease (relative risk = 1.61 [95% CI 1.59 to 1.64]), of ischaemic stroke (relative risk = 1.77 [95% CI 1.69 to 1.86]), and of haemorrhagic stroke (relative risk = 1.60 [95% CI 1.50 to 1.71]). By contrast, only modest increases in risks were seen in women who had hypertension in pregnancy but not in middle age (relative risks [95% CI] for coronary heart disease, ischaemic and haemorrhagic stroke: 1.17 [1.14 to 1.19], 1.18 [1.11 to 1.25], and 1.09 [1.02 to 1.18], respectively). Women with a history of hypertension both in pregnancy and in middle age had risks either similar to (haemorrhagic stroke) or modestly higher than (coronary heart disease and ischaemic stroke) those for hypertension in middle age alone. Similar patterns of risk of vascular deaths were seen across these subgroups (Table 4).

In women with information on blood pressure measured around nine years after recruitment (N = 3694), systolic blood pressure and diastolic blood pressure were both elevated in women with hypertension in pregnancy, whether or not they reported being treated for hypertension at study baseline (Table 5). In women with lipid profile assessment (N = 18,104), mean plasma concentrations of both total and HDL-cholesterol, as well as apolipoprotein B and A_1_, were lowest in women who were taking anti-hypertensive medication at baseline (*Web-only supplementary Table A*). For those who were not on anti-hypertensive treatment, those with a history of hypertension during pregnancy had lower total and HDL-cholesterol, as well as apolipoprotein B and A_1_, concentrations than those without such history.

## 4 Discussion

In this large cohort of middle-aged women, hypertension during pregnancy was associated with subsequent increase in risk of vascular disease incidence and mortality. The risks were increased for both coronary heart and cerebrovascular disease, and the risk of stroke was somewhat higher for ischaemic than for haemorrhagic stroke. However, many of these women with a history of hypertensive pregnancy were also being treated for hypertension in their 50s and 60s, and the association with vascular disease risk was substantially greater for being hypertensive in middle age than for having a history of hypertension during pregnancy alone.

Hypertensive disorders in pregnancy are important causes of morbidity and mortality to the mother during the perinatal period [1], [2], [34], [35]. Moreover, mothers with pregnancies complicated by hypertension, such as gestational hypertension or pre-eclampsia, have been reported to have higher risks of later coronary heart [4], [5], [6], [8], [9], [10], [11], [12], [13], [14], [15], [16] and cerebrovascular disease [5], [7], [9], [10], [11], [12], [13], [16] than women with normotensive pregnancies, but many of these studies have mean ages of disease events occurrence at less than 50 years (*Web-only supplementary Tables B and C, and Figs. A and B*), when vascular disease is still relatively uncommon [17], [18]. Compared to women aged 30–49 years in Europe, mortality rates for vascular disease are reported to be three times higher at age 50–59 years, twenty times higher at 60 to 69 years, and 300 times higher at age ≥ 70 years [17]. This pattern is similarly seen in the US [18]. Although a number of studies have investigated associations between hypertensive disorders during pregnancy and vascular outcomes at around middle age [7], [10], [12], [15], [16] the numbers of disease events were relatively small (*Web-only supplementary Tables B and C, and Figs. A and B*), and the contribution of other risk factors at that age was not examined in detail. In our study, we have shown that in middle age, a history of hypertension during pregnancy is associated with increased coronary heart disease and stroke risks, and these associations are independent of concurrent lifestyle factors and other potential confounders. Our findings are broadly consistent with other studies that showed elevated risks not just for coronary heart disease but also for cerebrovascular disease in women with hypertensive pregnancies [5], [7], [9], [10], [11], [12], [13], [16]. Although one study reported findings based on few ischaemic stroke events [15], the long-term risk of haemorrhagic stroke has not been previously reported. In our study, we showed that both ischaemic and haemorrhagic stroke risks are increased in women with hypertension in pregnancy albeit the magnitude of the risk particularly of haemorrhagic stroke was quite small.

Others have reported that women with hypertensive disorders in pregnancy are at increased risk of high blood pressure many years after the index pregnancy [4], [36]. Our findings suggest that this relation persists into middle age, and could mediate the relation between hypertension during pregnancy and long-term vascular disease risk. For example, the relative risk of coronary heart disease associated with hypertension during pregnancy was 1.29 (95% CI 1.27 to 1.31) (Table 2) compared to those without such history. However, the relative risk was only modestly increased 1.17 (95% CI 1.14 to 1.19) in women with hypertension during pregnancy who were not on hypertension treatment at baseline (Table 3), suggesting that the risk associated with hypertension during pregnancy appears to be largely explained by hypertension in middle age. Dyslipidaemia may also play a mediating role [37], [38], but the patterns of cholesterol and apolipoprotein concentrations in our cohort do not seem to concur with the observed vascular disease risk estimates with hypertension during pregnancy and being treated for hypertension at study baseline. It is plausible that, at least in our cohort, hypertension appears to play a more important role than dyslipidaemia in mediating the excess vascular disease risk associated with hypertension during pregnancy.

### 4.1 Limitations

Our study relied on women reporting at the time of their recruitment any hypertension during pregnancy. Although maternal recall of such obstetric history has been reported to have high specificity, the sensitivity is low [39]. In our cohort, a relatively high proportion (26.2%) reported to have ever had high blood pressure during pregnancy than might be expected from the prevalence of clinically-defined hypertensive disorders of pregnancy. Our estimate could reflect the prevalence of hypertension during pregnancy throughout women's reproductive life as well as hypertensive pregnancies without the clinical features associated with conditions as gestational hypertension or pre-eclampsia. In the prospective Northern Finland Birth Cohort 1966 study (involving > 96% expected births in the area), mothers have had their blood pressure measured during maternity clinic visits (93% attended > 5 times) [15]. Findings from this study suggest that while the proportions of pre-eclampsia (≈ 2%) and gestational hypertension (≈ 8%) were low, the proportion of new-onset hypertension which did not meet the clinical criteria for these disorders was ≈ 15%, and these phenotypes were shown to be associated with long-term risk of cardiovascular disease. Overall, Männistö and colleagues reported that the proportion of hypertension during pregnancy in their cohort was ≈ 25% (≈ 32% if including chronic hypertension). Although we could not rule out recall bias, the proportion of women with hypertension during pregnancy in our study is probably not implausible.

We also did not examine vascular disease risk in relation to specific types of hypertensive disorders in pregnancy as such information was not available. These different types may have varying effect sizes, with more severe forms likely to be associated with greater magnitude of vascular disease risk [4], so our risk estimates may underestimate the actual risks associated with some of these specific hypertensive disorders in pregnancy. It is also possible that some of the vascular disease events which occurred prior to study participation may be related to hypertension during pregnancy. However, differential reporting of obstetric history may exist between those with and without prevalent vascular disease so we excluded those with known vascular disease at baseline from the analysis. We adjusted for a number of potential confounders, but as an observational study, we could not rule out residual confounding. Nevertheless, our study is based on a large number of vascular disease events, including stroke events according to subtype, allowing us to examine in detail the associations with different vascular disease phenotypes at ages when the vascular disease burden in women is high.

### 4.2 Conclusion

In this large cohort of middle-aged women, hypertension during pregnancy was associated with increased risks of coronary heart disease and stroke. However, the risk of vascular disease associated with being hypertensive in middle age was greater than that associated with a history of hypertension during pregnancy alone. Since many of the women with a history of hypertension during pregnancy were also hypertensive in their 50s and 60s, the associations between history of hypertensive pregnancy with coronary heart disease and stroke are likely to be explained by increased blood pressure in middle age. Controlling hypertension is likely to be beneficial to middle-aged women, including those with hypertension during pregnancy. But efforts to prevent women with hypertensive pregnancy from developing hypertension by middle age may be just as important to help reduce their risk of developing vascular disease in the long-term.

## Funding source

This research was supported by Cancer Research UK [Grant C570/A16491], Medical Research Council (Grant MR/K02700X/1), and Oxford University BHF Centre of Research Excellence [British Heart Foundation grant RE/13/1/30181 to B.J.C.].

## Conflict of interest

None.

## Author contributions

VB, GR, and JG were involved in the conception, design of the study, and acquisition of data for the Million Women Study. DC, AB, and VB analysed the data. DC, BJC, AB, FLW, AK, JG, VB, and GR interpreted the data. DC drafted the first version of the manuscript. BJC, AB, FLW, AK, JG, VB, and GR gave critical intellectual input, and contributed in drafting revised versions of the manuscript. All authors gave their final approval of the version to be published.

## Figures and Tables

**Table 1:** Characteristics of parous women, by reported history of hypertension during pregnancy.

	Hypertension during pregnancy		All parous women N = 1,105,568
	No N = 815,560	Yes N = 290,008	
*Characteristics at baseline*			
Age (years), mean (SD)	56.0 (4.8)	55.9 (4.7)	56.0 (4.8)
Body mass index (kg/m^2^), mean (SD)	25.8 (4.4)	27.3 (5.1)	26.2 (4.6)
Age at menarche (years), mean (SD)	13.1 (1.6)	12.8 (1.6)	13.0 (1.6)
Parity, mean (SD)	2.4 (1.0)	2.4 (1.1)	2.4 (1.0)
% Current smoker (N)	21.9 (168,229)	17.6 (47,952)	20.8 (216,173)
% Non-drinker of alcoholic beverages (N)	28.9 (187,388)	30.3 (69,220)	29.3 (256,608)
% Engage in vigorous exercise ≤ 1 per week (N)	47.9 (376,209)	48.2 (134,897)	48.0 (511,106)
% Lowest third of socioeconomic status (N)	32.8 (265,143)	32.4 (93,354)	32.7 (358,497)
% Ever use of hormone replacement therapy (N)	51.4 (414,502)	49.7 (142,411)	51.0 (556,913)
% Being treated for hypertension (N)	10.1 (82,145)	27.3 (79,163)	14.6 (161,308)
% Being treated for diabetes (N)	1.7 (14,164)	3.0 (8646)	2.1 (22,810)
% Being treated for hypercholesterolaemia (N)	2.3 (18,552)	3.5 (10,126)	2.6 (28,678)

*Follow-up details*			
First vascular disease event (ICD-10), no. (person-years [1000s])			
Coronary heart disease (I20 to I25)	46,580 (9449)	21,581 (3333)	68,161 (12,782)
Cerebrovascular disease (I60 to I69)	16,226 (9600)	6771 (3408)	22,997 (13,008)
Ischaemic stroke (I63)	5784 (9635)	2581 (3422)	8365 (13,057)
Haemorrhagic stroke (I60 to I62)	4164 (9641)	1538 (3426)	5702 (13,067)
Underlying cause of death (ICD-10), no. (person-years [1000s])			
Coronary heart disease (I20 to I25)	5216 (11,850)	2520 (4219)	7736 (16,068)
Cerebrovascular disease (I60 to I69)	4032 (11,850)	1522 (4219)	5554 (16,068)

ICD-10 — International Classification of Diseases, 10th Revision.

**Table 2:** Relative risks (95% confidence interval) of vascular disease in parous women, by reported history of hypertension during pregnancy.

Vascular disease (ICD-10)	No. with vascular disease outcome		Relative risk (95% confidence interval)
	Hypertension during pregnancy		
	Yes N = 290,008	No N = 815,560	
First vascular disease event			
Coronary heart disease (I20 to I25)	21,581	46,580	1.29 (1.27 to 1.31)
Cerebrovascular disease (I60 to I69)	6771	16,226	1.23 (1.20 to 1.27)
Ischaemic stroke (I63)	2581	5784	1.29 (1.23 to 1.35)
Haemorrhagic stroke (I60 to I62)	1538	4164	1.14 (1.07 to 1.21)
Underlying cause of death			
Coronary heart disease (I20 to I25)	2520	5216	1.35 (1.29 to 1.42)
Cerebrovascular disease (I60 to I69)	1522	4032	1.16 (1.09 to 1.23)

ICD-10 — International Classification of Diseases, 10th Revision; relative risks based on Cox regression with age as the underlying time variable; risk estimates stratified by region of recruitment, and adjusted for smoking, socioeconomic status, exercise, alcohol intake, body mass index, parity, ever use of hormone replacement therapy, treatment for diabetes, and treatment for hypercholesterolaemia at baseline.

**Table 3:** Relative risk (95% group-specific confidence interval [g-sCI]) of incident coronary heart disease and stroke in parous women, by reported history of hypertension during pregnancy and treatment for hypertension at baseline.

Incident vascular disease (ICD-10)	Hypertension during pregnancy	Being treated for hypertension at baseline	No. with outcome	Relative risk (95% g-sCI)
*Coronary heart disease (I20 to I25)*				
	No	No	33,884	1.00 (0.99 to 1.01)
	Yes	No	8999	1.17 (1.14 to 1.19)
	No	Yes	12,696	1.61 (1.59 to 1.64)
	Yes	Yes	12,582	1.78 (1.76 to 1.82)

*Ischaemic stroke (I63)*				
	No	No	4077	1.00 (0.97 to 1.03)
	Yes	No	1056	1.18 (1.11 to 1.25)
	No	Yes	1707	1.77 (1.69 to 1.86)
	Yes	Yes	1525	1.86 (1.76 to 1.96)

*Haemorrhagic stroke (I60 to I62)*				
	No	No	3184	1.00 (0.96 to 1.04)
	Yes	No	760	1.09 (1.02 to 1.18)
	No	Yes	980	1.60 (1.50 to 1.71)
	Yes	Yes	778	1.48 (1.38 to 1.59)

ICD-10 — International Classification of Diseases, 10th Revision; relative risks are hazard ratios estimated using Cox regression with age as the underlying time variable, stratified by region of recruitment, and adjusted for smoking, socioeconomic status, exercise, alcohol intake, body mass index, parity, ever use of hormone replacement therapy, treatment for diabetes, and treatment for hypercholesterolaemia at baseline; reference group: no history of hypertensive pregnancy and not on anti-hypertensive treatment at baseline.

**Table 4:** Relative risk (95% group-specific confidence interval [g-sCI]) of coronary heart and cerebrovascular disease mortality in parous women, by reported history of hypertension during pregnancy and treatment for hypertension at baseline.

Underlying cause of death (ICD-10)	Hypertension during pregnancy	Being treated for hypertension at baseline	No. with outcome	Relative risk (95% g-sCI)
*Coronary heart disease (I20 to I25)*				
	No	No	3582	1.00 (0.97 to 1.04)
	Yes	No	967	1.23 (1.16 to 1.31)
	No	Yes	1634	1.86 (1.77 to 1.96)
	Yes	Yes	1553	2.02 (1.92 to 2.14)

*Cerebrovascular disease (I60 to I69)*				
	No	No	2848	1.00 (0.96 to 1.04)
	Yes	No	658	1.10 (1.02 to 1.18)
	No	Yes	1184	1.88 (1.78 to 1.99)
	Yes	Yes	864	1.69 (1.58 to 1.81)

ICD-10 — International Classification of Diseases, 10th Revision; relative risks are hazard ratios estimated using Cox regression with age as the underlying time variable, stratified by region of recruitment, and adjusted for smoking, socioeconomic status, exercise, alcohol intake, body mass index, parity, ever use of hormone replacement therapy, treatment for diabetes, and treatment for hypercholesterolaemia at baseline; reference group: no history of hypertensive pregnancy and not on anti-hypertensive treatment at baseline.

**Table 5:** Mean blood pressure (95% confidence interval [CI]) at resurvey in 3694 women, by reported history of hypertension during pregnancy and treatment of hypertension at baseline.

Hypertension during pregnancy	Being treated for hypertension at baseline	No. of women	Mean systolic blood pressure (mm Hg) at resurvey (95% CI)	Mean diastolic blood pressure (mm Hg) at resurvey (95% CI)
No	No	2636	132.9 (132.3 to 133.6)	77.3 (77.0 to 77.7)
Yes	No	631	137.9 (136.7 to 139.2)	79.9 (79.2 to 80.6)
No	Yes	247	139.7 (137.7 to 141.7)	80.3 (79.1 to 81.5)
Yes	Yes	180	139.0 (136.6 to 141.3)	79.5 (78.2 to 80.9)

Mean blood pressure adjusted for age at blood pressure measurement and region of recruitment; resurvey at nine years, on average, after baseline recruitment.

## Appendix A Supplementary data

Supplementary material. Image 1

## References

[1] WHO Study Group (1987). The Hypertensive Disorders of Pregnancy. Technical Series Report 785.

[2] Creanga A.A., Berg C.J., Syverson C., Seed K., Bruce F.C., Callaghan W.M. (2015). Pregnancy-related mortality in the United States, 2006–2010. Obstet. Gynecol..

[3] Ananth C.V., Peedicayil A., Savitz D.A. (1995). Effect of hypertensive diseases in pregnancy on birthweight, gestational duration, and small-for-gestational-age births. Epidemiology.

[4] Bellamy L., Casas J.P., Hingorani A.D., Williams D.J. (2007). Pre-eclampsia and risk of cardiovascular disease and cancer in later life: systematic review and meta-analysis. BMJ.

[5] Hannaford P., Ferry S., Hirsch S. (1997). Cardiovascular sequelae of toxaemia of pregnancy. Heart.

[6] Smith G.C., Pell J.P., Walsh D. (2001). Pregnancy complications and maternal risk of ischaemic heart disease: a retrospective cohort study of 129,290 births. Lancet.

[7] Wilson B.J., Watson M.S., Prescott G.J. (2003). Hypertensive diseases of pregnancy and risk of hypertension and stroke in later life: results from cohort study. BMJ.

[8] Wikstrom A.K., Haglund B., Olovsson M., Lindeberg S.N. (2005). The risk of maternal ischaemic heart disease after gestational hypertensive disease. BJOG.

[9] Ray J.G., Vermeulen M.J., Schull M.J., Redelmeier D.A. (2005). Cardiovascular health after maternal placental syndromes (CHAMPS): population-based retrospective cohort study. Lancet.

[10] Arnadottir G.A., Geirsson R.T., Arngrimsson R., Jonsdottir L.S., Olafsson O. (2005). Cardiovascular death in women who had hypertension in pregnancy: a case–control study. BJOG.

[11] Lykke J.A., Langhoff-Roos J., Sibai B.M., Funai E.F., Triche E.W., Paidas M.J. (2009). Hypertensive pregnancy disorders and subsequent cardiovascular morbidity and type 2 diabetes mellitus in the mother. Hypertension.

[12] Bhattacharya S., Prescott G.J., Iversen L., Campbell D.M., Smith W.C., Hannaford P.C. (2012). Hypertensive disorders of pregnancy and future health and mortality: a record linkage study. Pregnancy Hypertens..

[13] Skjærven R., Wilcox A.J., Klungsoyr K. (2012). Cardiovascular mortality after pre-eclampsia in one child mothers: prospective, population based cohort study. BMJ.

[14] Zhao H.Y., Chen X.W., Niu J.Q. (2012). History of pregnancy induced hypertension is linked with increased risk of cardio-cerebral vascular events. Zhonghua Xin Xue Guan Bing Za Zhi.

[15] Männistö T., Mendola P., Vääräsmäki M. (2013). Elevated blood pressure in pregnancy and subsequent chronic disease risk. Circulation.

[16] Heida K.Y., Franx A., van Rijn B.B. (2015). Earlier age of onset of chronic hypertension and type 2 diabetes mellitus after a hypertensive disorder of pregnancy or gestational diabetes mellitus. Hypertension.

[17] World Health Organization (2000-2012). Global health estimates 2014 summary tables: deaths by cause, age and sex, by WHO region. http://www.who.int/healthinfo/global_burden_disease/en/.

[18] Centers for Disease Control and Prevention, National Center for Health Statistics. Underlying cause of death (1999-2013). CDC WONDER online database, released 2015, from the Multiple Cause of Death Files, 1999–2013, as compiled from data provided by the 57 vital statistics jurisdictions through the Vital Statistics Cooperative Program. http://wonder.cdc.gov/ucd-icd10.html.

[19] Mozaffarian D., Benjamin E.J., Go A.S. (2016). Heart disease and stroke statistics — 2016 update: a report from the American Heart Association. Circulation.

[20] Townsend N., Williams J., Bhatnagar P., Wickramasinghe K., Rayner M. (2014). Cardiovascular Disease Statistics 2014.

[21] The Million Women Study http://www.millionwomenstudy.org/introduction/.

[22] Health and Social Care Information Centre http://www.hscic.gov.uk.

[23] National Records of Scotland NHS Central Register. http://www.nrscotland.gov.uk/statistics-and-data/nhs-central-register.

[24] Health and Social Care Information Centre Hospital Episode Statistics. http://www.hscic.gov.uk/hes.

[25] Administrative Data Liaison Service Scottish Morbidity Records. http://www.adls.ac.uk/nhs-scotland/scottish-morbidity-database-smr/?detail.

[26] World Health Organization International statistical classification of diseases and related health problems 10th revision. http://apps.who.int/classifications/icd10/.

[27] Wright F.L., Green J., Canoy D. (2012). Vascular disease in women: comparison of diagnoses in hospital episode statistics and general practice records in England. BMC Med. Res. Methodol..

[28] Canoy D., Beral V., Balkwill A. (2015). Age at menarche and risks of coronary heart and other vascular diseases in a large UK cohort. Circulation.

[29] Townsend P., Phillimore P., Beattie A. (1988). Health and Deprivation: Inequality and the North.

[30] Plummer M. (2004). Improved estimates of floating absolute risk. Stat. Med..

[31] Stata MP (2013). 13.0. College Station.

[32] Jasper A.M. (2016). Jasper Makes Plots, R Package Version 2-210.

[33] R Development Core Team (2016). R: A Language and Environment for Statistical Computing.

[34] Kuklina E.V., Ayala C., Callaghan W.M. (2009). Hypertensive disorders and severe obstetric morbidity in the United States. Obstet. Gynecol..

[35] Bramham K., Parnell B., Nelson-Piercy C., Seed P.T., Poston L., Chappell L.C. (2014). Chronic hypertension and pregnancy outcomes: systematic review and meta-analysis. BMJ.

[36] Garovic V.D., August P. (2013). Preeclampsia and the future risk of hypertension: the pregnant evidence. Curr. Hypertens. Rep..

[37] Magnussen E.B., Vatten L.J., Smith G.D., Romundstad P.R. (2009). Hypertensive disorders in pregnancy and subsequently measured cardiovascular risk factors. Obstet. Gynecol..

[38] Fanshawe A.E., Ibrahim M. (2013). The current status of lipoprotein (a) in pregnancy: a literature review. J. Cardiol..

[39] Stuart J.J., Bairey Merz C.N., Berga S.L. (2013). Maternal recall of hypertensive disorders in pregnancy: a systematic review. J. Women's Health (Larchmt).

