# Supplementary material for: Hypertension in pregnancy and risk of coronary heart disease and stroke: A prospective study in a large UK cohort
Source: Int J Cardiol. 2016 Nov 1;222:1012–8. doi: 10.1016/j.ijcard.2016.07.170 (PMC5047033; doi:10.1016/j.ijcard.2016.07.170)
Supplement: Supplementary file 1 — Supplementary material. [file mmc1.docx]

**Appendix A**. Supplementary data

# Hypertension in pregnancy and risk of coronary heart disease and stroke: a prospective study in a large UK cohort

D Canoy^a^, BJ Cairns^a^, A Balkwill^a^, FL Wright^a^, A Khalil^b^, V Beral^a^, J Green^a^, and G Reeves^a^ for the *Million Women Study Collaborators*

^a^Cancer Epidemiology Unit, Nuffield Department of Population Health, University of Oxford, Oxford, UK

^b^Department of Obstetrics and Gynaecology, St. George’s Medical School, University of London, London, UK

CONTENTS:

**Table A**. Mean plasma concentrations of cholesterol and lipid fractions at resurvey in 18,104 women, by history of hypertension during pregnancy and

treatment for hypertension at baseline.………………………………………………………………………………………………………..……………………………………………………………………….…………. *Page 2*

**Table B**. Prospective studies investigating the relation between hypertension during pregnancy and coronary heart disease risk……………………………………………………. *Page 3*

**Table C**. Prospective studies investigating the relation between hypertension during pregnancy and cerebrovascular disease risk………………………………………………….. *Page 5*

**Figure A**. Estimated women’s ages at the beginning and end of follow-up of prospective studies investigating the relation between hypertension

during pregnancy and coronary heart disease risk.……….………………………………………………………………………………………………………………………………………………………………….. *Page 7*

**Figure B**. Estimated women’s ages at the beginning and end of follow-up of prospective studies investigating the relation between hypertension

during pregnancy and cerebrovascular disease risk.………………………………………….……………………………………………………………………………………………………………………………… *Page 8*

**References**………………………………..……………………………………………………………………………………………………………………………………………………………………………………………………… *Page 9*

Table A. Mean plasma concentrations of cholesterol and lipid fractions (95% confidence interval) at resurvey in 18,104 women, by history of hypertension during pregnancy and treatment for hypertension at baseline.

| Hypertension during pregnancy | Being treated for hypertension at baseline | No. of women | Total cholesterol (mmol/L) | HDL-cholesterol (mmol/L) | Apolipoprotein B (g/L) | Apolipoprotein A_1_ (g/L) | Apolipoprotein B to A_1_ ratio |
| --- | --- | --- | --- | --- | --- | --- | --- |
|  |  |  |  | Mean concentration (95% CI) at resurvey | | |  |
| No | No | 12,888 | 5.90 (5.87 to 5.91) | 1.64 (1.63 to 1.64) | 1.08 (1.07 to 1.08) | 1.72 (1.71 to 1.72) | 0.64 (0.64 to 0.65) |
| Yes | No | 3129 | 5.79 (5.75 to 5.83) | 1.59 (1.57 to 1.60) | 1.06 (1.06 to 1.07) | 1.69 (1.68 to 1.70) | 0.65 (0.64 to 0.65) |
| No | Yes | 1167 | 5.35 (5.29 to 5.41) | 1.49 (1.46 to 1.51) | 0.99 (0.98 to 1.01) | 1.65 (1.63 to 1.66) | 0.62 (0.61 to 0.63) |
| Yes | Yes | 920 | 5.35 (5.27 to 5.42) | 1.46 (1.43 to 1.49) | 1.00 (0.99 to 1.02) | 1.63 (1.61 to 1.65) | 0.63 (0.62 to 0.64) |

Mean lipid concentration adjusted for age at blood collection and region of recruitment; Resurvey at nine years, on average, after baseline recruitment.

Table B. Prospective studies investigating the relation between hypertension during pregnancy and coronary heart disease risk.

| Author / Country / Year | Study population | Type of exposure | Source of exposure data | No. of participants | No. of events | Year of study entry & exit | Mean follow-up duration from index pregnancy or study (years) | Mean age (years) at baseline [at outcome] | Relative risk | Comments |
| --- | --- | --- | --- | --- | --- | --- | --- | --- | --- | --- |
| **Study baseline period: At index pregnancy** | | |  |  |  |  |  |  |  |  |
| Männistö (Finland); 2013 | Mothers of offspring in a population-based birth cohort | Chronic & new-onset hypertension (e.g. isolated, gestational & pre-eclampsia) | Questionnaires at maternity clinic & linkage with antenatal records | 10,314 | 1875 incident CHD; 778 incident MI; 206 MI deaths | 1966 until ≈2006 | Mean= 39.4 | ≈26 [66.7] | From 1.26 to 5.12 (higher RRs for more severe exposure and outcome phenotypes) | Reference group: normotensive pregnancies; RR adjusted for perinatal factors |
| Skjærven (Norway); 2012 | Mothers with deliveries recorded in a national birth registry | Pre-eclampsia | Retrospective review of birth registry | 836,147 | 1086 CHD deaths | 1967 to 2002 until 2009 | Median= 25 | 24.3 [49.1*] | 1.7 to 9.3, with higher risks for preterm births and lower risks for ≥2 lifetime pregnancies | Reference group: ≥2 lifetime pregnancies & no pre-eclampsia; RR adjusted for perinatal & reproductive factors |
| Bhatta-charya (Scotland); 2012 | Mothers with deliveries recorded in population-based birth registry | Gestational hypertension & pre-eclampsia (including eclampsia) | Retrospective review of maternity and birth registers | 34,854 | 733 CHD deaths; 2810 incident CHD | From 1950 to 2008 | Up to 58 years (mean ≈40) | ≈24 [≈64 at death] | For pre-eclampsia, 1.18 (NS) for incident CHD, 1.38 for CHD death; for gestational hypertension, 1.22 for incident CHD, 1.35 for CHD death | Hospitalisations not modelled as incident outcomes; Risk estimates are odds ratios |
| Lykke (Denmark); 2009 | Mothers with deliveries recorded in a national birth registry | Gestational hypertension & pre-eclampsia | Retrospective review of birth registry | 782,287 | 8516 incident CHD | 1978 to 2007 until 2008 | Mean= 14.6 | 26.8 [41.6] | From 1.48 to 1.61; higher RRs with pregnancy complications & repeated pre-eclampsia | Reference group: no hypertensive pregnancy; RR adjusted for perinatal factors (but not for body size or smoking during pregnancy) |
| Arnadottir (Iceland); 2005 | Mothers with deliveries recorded in a single hospital (undefined catchment area) | Chronic hypertension, gestational hypertension, pre-eclampsia & eclampsia | Retrospective review of maternity records | 325 exposed, 629 unexposed | 171 CHD deaths | 1931 to 1947 until 1996 | Median= 50 (exposed group), 55 (unexposed group) | Either ages not mentioned | 1.66; Unadjusted CHD death rates increased with pre-eclampsia severity (p=0.02); NS for chronic hypertension only | Exposed group matched for date of delivery, parity, and age at pregnancy; RR unadjusted for other covariates |
| Ray (Canada); 2005 | Nulliparous mothers with deliveries recorded in province-wide health insurance database | Maternal placental syndrome | Retrospective review of health insurance database | 1,026,265 | 1440 incident CHD | From 1990 to 2004 | Median= 8.7 | 28 [38.3] | 2.0 (1.7 to 2.3) | Reference group: No maternal placental syndrome; RR adjusted for perinatal factors and medical history including gestational diabetes, & history of hypertension or dyslipidaemia |
| Wikstrom (Sweden); 2005 | Mothers with deliveries recorded in a national birth registry | Gestational hypertension & pre-eclampsia | Retrospective review of national birth registry | 403,550 | 2579 incident CHD | 1973 to 1982, followed from 1987 to 2001 | 19 to 28 (at least 15 years) | 85% within 25 to 44 [Median= 48] | From 1.6 to 2.8; RR increases in relation to parity and co-occurring pregnancy complications | Included only those without diabetes or chronic hypertension during pregnancy; Reference group: Non-hypertensive first pregnancy; RR adjusted for some perinatal factors |
| Wilson (Scotland); 2003 | Mothers with deliveries recorded in population-based birth registry, & traced for assessment for this study | Gestational hypertension & pre-eclampsia | Retrospective review of maternity and birth registers | 3593 | 138 incident CHD, 53 CHD deaths | 1951 to 1970 for events occurring between 1980 & 1999 | Not mentioned | 24 [possibly between 53 and 68] | NS | No prior hypertension during index pregnancy; Reference group: No hypertension during pregnancy; Exposed group matched by age at delivery & birth year to unexposed group; RR adjusted for some perinatal factors |
| Smith (Scotland); 2001 | Nulliparous mothers with deliveries are recorded in national health database | Pre-eclampsia | Retrospective review of maternity health records | 129,920 | 313 incident CHD, 43 CHD deaths | 1981 to 1985 until 1999 | 15 to 19 | Median= 23 [Median= 38.5] | Incident CHD= 2.0, CHD death= 1.7 (CHD death); RRs higher with pregnancy complications | Reference group: Non-hypertensive pregnancy with term delivery & within highest four birth weight fifths; RR adjusted for perinatal factors except smoking |
| **Study baseline period: Outside of index pregnancy** | | |  |  |  |  |  |  |  |  |
| Heida (Nether-lands); 2015 | Parous women who attended health checks in two cohort studies | History of hypertension during pregnancy | Response to study baseline questionnaire | 22,265 | 1478 incident CHD | From 1993 to 1997 until 1 January 2008 | Median=13 | 53.7 (29 at first pregnancy) [≈68] | 1.28 (1.16 if adjusted for baseline blood pressure) | Excludes type 2 diabetes, hypertension or cardiovascular disease prior to age of first pregnancy |
| Zhao (China); 2012 | Mothers with no prior CVD & attended a health check who previously delivered at a hospital (undefined catchment area) | Pregnancy-induced hypertension | Retrospective review of hospital records | 4630 | 29 incident MI | 2006 to 2007 until 2010 (but index pregnancies from 1976 to 2008) | From 2 to 34 years from index pregnancy) | 27.4 at index pregnancy; 42 (exposed group) & 38 (unexposed group) at baseline [likely <50] | 3.91 | Reference group: No history of hypertensive pregnancy; RR adjusted for age at perinatal and other factors including blood pressure, lipids & body mass index measured at health exam |
| Hannaford (UK): 1997 | Non-users of pill in the RCGP Oral Contraception Study | History of toxaemia of pregnancy | Study forms and clinic records filled in by general practitioners | 23,000 | 285 incident CHD | 1968 to 1969 until 1995 | Up to 26 (214,356 women-years) | 29 [Possibly in their 40s] | 1.65; subsequent hypertension after baseline: No= NS, Yes= 1.59 | Never-users of oral contraceptive pill; RR adjusted for age, smoking, social class |

RR – relative risk; AOG – age of gestation; CHD – coronary heart disease; CVD – cardiovascular disease; MI – myocardial infarction; NS – not significant; Follow-up for outcomes usually inclusive of study time period except Wikstrom 2005; Incident CHD usually refers to first CHD hospitalisation or death except Ray 2005 (hospitalisations and/or revascularisations only) and Bhattacharya 2012 (hospitalisations only); *Information obtained by personal communication (Skjærven 2014 and Heida 2015).

Table C. Prospective studies investigating the relation between hypertension during pregnancy and cerebrovascular disease risk.

| Author (Country); Year | Study population | Type of exposure | Source of exposure data | No. of participants | No. of events | Year of study entry & exit | Follow-up duration from index pregnancy or study (years) | Mean age (years) at baseline [at outcome] | Relative risk | Comments |
| --- | --- | --- | --- | --- | --- | --- | --- | --- | --- | --- |
| **Study baseline period: At index pregnancy** | | |  |  |  |  |  |  |  |  |
| Männistö (Finland); 2013 | Mothers of offspring in a population-based birth cohort | Chronic & new-onset hypertension (e.g. isolated, gestational & pre-eclampsia) | Questionnaires at maternity clinic & linkage with antenatal records | 10,314 | 595 incident ischaemic stroke | 1966 until ≈2006 | Mean= 39.4 | ≈26 [66.7] | NS except gestational hypertension (1.59 and chronic hypertension (1.80) | Reference group: normotensive pregnancies; RR adjusted for perinatal factors |
| Skjærven (Norway); 2012 | Mothers with deliveries recorded in a national birth registry | Pre-eclampsia | Retrospective review of birth registry | 836,147 | 1119 CeVD deaths | 1967 to 2002 until 2009 | Median= 25 | 24.3 [49.1*] | One lifetime pregnancy: 2.1 to 10.4 with higher RR for preterm births; NS for pre-eclampsia involving ≥2 lifetime pregnancies | Reference group: ≥2 lifetime pregnancies & no pre-eclampsia; RR adjusted for perinatal & reproductive factors |
| Bhatta-charya (Scotland); 2012 | Mothers with deliveries recorded in population-based birth registry | Gestational hypertension & pre-eclampsia (including eclampsia) | Retrospective review of maternity and birth registers | 34,854 | 266 CeVD deaths; 1459 incident CeVD | From 1950 to 2008 | Up to 58 years (mean ≈40) | ≈24 [≈64 at death] | NS for pre-eclampsia; for gestational hypertension, NS for incident CeVD, 1.28 for CeVD death | Hospitalisations not modelled as incident outcomes; Risk estimates are odds ratios |
| Lykke (Denmark); 2009 | Mothers with deliveries recorded in a national birth registry | Gestational hypertension & pre-eclampsia | Retrospective review of birth registry | 782,287 | 8987 incident CeVD | 1978 to 2007 until 2008 | Mean= 14.6 | 26.8 [41.6] | From 1.43 to 1.58; higher RR with pregnancy complications & repeated pre-eclampsia | Reference group: no hypertensive pregnancy; RR adjusted for perinatal factors (but not for body size or smoking during pregnancy) |
| Arnadottir (Iceland); 2005 | Mothers with deliveries recorded in a single hospital (undefined catchment area) | Chronic hypertension, gestational hypertension, pre-eclampsia & eclampsia | Retrospective review of maternity records | 325 exposed, 629 unexposed | 72 CeVD deaths | 1931 to 1947 until 1996 | Median= 50 (exposed group), 55 (unexposed group) | Either ages not mentioned | NS overall, but 8.4 for aged ≤64 years (NS for older ages) | Exposed matched for date of delivery, parity, and age at pregnancy; RR unadjusted for other covariates |
| Ray (Canada); 2005 | Nulliparous mothers with deliveries recorded in province-wide health insurance database | Maternal placental syndrome | Retrospective review of health insurance database | 1,026,265 | 415 incident CeVD | From 1990 to 2004 | Median= 8.7 | 28 [38.3] | 2.0 (1.7 to 2.3) | Reference group: No maternal placental syndrome; RR adjusted for perinatal factors and medical history including gestational diabetes, & history of hypertension or dyslipidaemia |
| Wilson (Scotland); 2003 | Mothers with deliveries recorded in population-based birth registry, & traced for assessment for this study | Gestational hypertension & pre-eclampsia | Retrospective review of maternity and birth registers | 3593 | 69 incident CeVD, 34 CeVD deaths | 1951 to 1970 for events occurring between 1980 & 1999 | Not mentioned | 24 [possibly between 53 and 68] | Gestational hypertension: NS for both outcomes; Pre-eclampsia or eclampsia: from 2.10 (incident CeVD) to 3.59 (CeVD deaths) | No prior hypertension during index pregnancy; Reference group: No hypertension during pregnancy; Exposed group matched by age at delivery & birth year to unexposed group; RR adjusted for some perinatal factors |
| **Study baseline period: Outside of index pregnancy** | | |  |  |  |  |  |  |  |  |
| Heida (Nether-lands); 2015 | Parous women who attended health checks in two cohort studies | History of hypertension during pregnancy | Response to study baseline questionnaire | 22,265 | 720 incident stroke | From 1993 to 1997 until 1 January 2008 | Median=13 | 53.7 (29 at first pregnancy) [≈68] | 1.26 (NS if adjusted for baseline blood pressure) | Excludes type 2 diabetes, hypertension or cardiovascular disease prior to age of first pregnancy; ischaemic not differentiated from haemorrhagic subtype |
| Hannaford (UK): 1997 | Non-users of pill in the RCGP Oral Contraception Study | History of toxaemia of pregnancy | Study forms and clinic records filled in by general practitioners | 23,000 | 118 incident CeVD | 1968 to 1969 until 1995 | Up to 26 (214,356 women-years) | 29 [Possibly in their 40s] | NS (overall and when stratified by subsequent hypertension after baseline) | Never-users of oral contraceptive pill; RR adjusted for age, smoking, social class |

RR – relative risk; AOG – age of gestation; CeVD – cerebrovascular disease; CVD – cardiovascular disease; MI – myocardial infarction; NS – not significant; Follow-up for outcomes usually inclusive of study time period; Incident CeVD usually refers to first CeVD hospitalisation or death except Ray 2005 (hospitalisations and/or revascularisations only); *Information obtained by personal communication (Skjærven 2014 and Heida 2015).

Figure A. Estimated women’s ages (mean or median) at the beginning and end of follow-up of prospective studies investigating the relation between hypertension during pregnancy and coronary heart disease risk. Numbers on bars are numbers of disease events. Studies in dotted lines are those with follow-up up to around middle age.

Figure B. Estimated women’s ages (mean or median) at the beginning and end of follow-up of prospective studies investigating the relation between hypertension during pregnancy and cerebrovascular disease risk. Numbers on bars are numbers of disease events. Studies in dotted lines are those with follow-up up to around middle age.

References

Arnadottir GA, Geirsson RT, Arngrimsson R, Jonsdottir LS, Olafsson O. Cardiovascular death in women who had hypertension in pregnancy: a case-control study. BJOG 2005;112:286-92.

Bhattacharya S, Prescott GJ, Iversen L, Campbell DM, Smith WC, Hannaford PC. Hypertensive disorders of pregnancy and future health and mortality: A record linkage study. Pregnancy Hypertens 2012;2:1-7.

Hannaford P, Ferry S, Hirsch S. Cardiovascular sequelae of toxaemia of pregnancy. Heart 1997;77:154-8

Heida KY, Franx A, Van Rijn BB, Eijkemans MJ, Boer JM, Verschuren MW, Oudijk MA, Bots ML, Van Der Schouw YT. Earlier age of onset of chronic hypertension and Type 2 diabetes mellitus after a hypertensive disorder of pregnancy or gestational diabetes mellitus. Hypertension 2015;66:1116-22.

Lykke JA, Langhoff-Roos J, Sibai BM, Funai EF, Triche EW, Paidas MJ. Hypertensive pregnancy disorders and subsequent cardiovascular morbidity and type 2 diabetes mellitus in the mother. Hypertension 2009;53:944-51.

Männisto T, Mendola P, Vääräsmäki M, Järvelin MR, Hartikainen AL, Pouta A, Suvanto E. Elevated blood pressure in pregnancy and subsequent chronic disease risk. Circulation 2013;127:681-90.

Ray JG, Vermeulen MJ, Schull MJ, Redelmeier DA. Cardiovascular health after maternal placental syndromes (CHAMPS): population-based retrospective cohort study. Lancet 2005;366:1797-803.

Skjærven R, Wilcox AJ, Klungsoyr K, Irgens LM, Vikse BE, Vatten LJ, Lie RT. Cardiovascular mortality after pre-eclampsia in one child mothers: prospective, population based cohort study. BMJ 2012;345:e7677.

Smith GC, Pell JP, Walsh D. Pregnancy complications and maternal risk of ischaemic heart disease: a retrospective cohort study of 129,290 births. Lancet 2001;357:2002-6.

Wikstrom AK, Haglund B, Olovsson M, Lindeberg SN. The risk of maternal ischaemic heart disease after gestational hypertensive disease. BJOG 2005;112:1486-91.

Wilson BJ, Watson MS, Prescott GJ, Sunderland S, Campbell DM, Hannaford P, Smith WC. Hypertensive diseases of pregnancy and risk of hypertension and stroke in later life: results from cohort study. BMJ 2003;326:845.

Zhao HY, Chen XW, Niu JQ, Hou GS, Sun J, Jin C, Gao JS, Zheng XM, Wu SL. [History of pregnancy induced hypertension is linked with increased risk of cardio-cerebral vascular events]. Zhonghua Xin Xue Guan Bing Za Zhi 2012;40:645-51.
